# The RF Cap: A 26-channel flexible RF coil cap for optimized concurrent TMS/fMRI experiments at 3T

**DOI:** 10.1162/IMAG.a.922

**Published:** 2025-10-14

**Authors:** Lucia I. Navarro de Lara, Sebastian Ardila, Lincoln Craven-Brightman, Evgenii Kim, Mohammad Daneshzand, Lawrence L. Wald, Jyrki Ahveninen, Jason P. Stockmann, Aapo Nummenmaa

**Affiliations:** Athinoula A. Martinos Center for Biomedical Imaging, Department of Radiology, Massachusetts General Hospital, Charlestown, MA, United States; Harvard Medical School, Boston, MA, United States

**Keywords:** concurrent TMS/fMRI, MRI of neuromodulation, flexible RF coils

## Abstract

Combining brain imaging methods with non-invasive brain stimulation such as transcranial magnetic stimulation (TMS) is a rapidly expanding field with the potential to drastically improve the understanding of brain function. However, currently there is no generally applicable hardware solution optimized for these types of acquisitions. To make concurrent TMS/fMRI experiments at 3 T feasible without sacrificing imaging quality, we have designed, constructed, and tested the first of its kind “RF Cap”: a 26-channel flexible RF coil cap. The RF Cap achieves full brain coverage with high sensitivity while allowing the administration of TMS at most targets over the scalp with an easy setup and possibility of using a neuronavigation system. The RF Cap consists of a FLEXIBLE and a RIGID part. The FLEXIBLE part is a neoprene cap with 26 flexible RF coaxial cable loops sewn onto it and distributed following a soccer ball layout. The RF elements were interfaced using flexible PCBs and incorporating a BALUN to minimize common modes on the short cables connecting the elements to their preamplifiers placed on the RIGID part. This solution provides a user-friendly approach for concurrent TMS/fMRI acquisitions while ensuring optimal patient comfort. We show that the RF Cap offers at least 4 times more SNR than a birdcage coil at the center of the brain and 10–16 times more SNR on the cortex. The effects of the TMS on the SNR of the RF Cap are between 10% and 25% loss over the region where the TMS coil is placed. The RF Cap has the potential to transform concurrent TMS/fMRI into a practical and useful neuroscientific tool as well as to pave the way for future clinical applications.

## 1 Introduction

The development of fMRI (Belliveau et al., 1991; Ogawa et al., 1990) was a milestone for the study of brain function non-invasively. Since then, the number of fMRI studies applied to the different brain research fields has grown rapidly along with the development of a wide range of methods to improve acquisition, analysis, and interpretation of the data. One of the breakthroughs in the improvement of the acquisition methods was the development of whole-head close-fitting “RF helmets” using receive-only surface coil multichannel arrays (Wiggins et al., 2006). This hardware together with parallel imaging methods (Breuer et al., 2009; Pruessmann et al., 1999) has enabled high-sensitivity fMRI with higher spatial resolution as well as reduced geometric distortion and signal loss during acquisition. The quality of today’s fMRI depends critically on these advances in dedicated RF receive coils developed in the past two decades (Keil et al., 2013; Wiggins et al., 2006).

Although BOLD fMRI has become a widely used powerful brain research tool, the causal relationships between activated regions are masked by the sluggishness of the hemodynamic response as well as the challenges to precisely activate different parts of the brain at desired time points using sensory stimuli or tasks only. One possibility to help dissect the causal relationships between the activated regions is combining non-invasive cortical brain stimulation techniques such as transcranial magnetic stimulation (TMS) (Barker et al., 1985) with fMRI. This combination of concurrent TMS-fMRI was presented for the first time more than 20 years ago (Bohning et al., 1999) and it holds potential for studying the causal relationships between the cortical and subcortical nodes of large-scale brain networks (Bestmann & Feredoes, 2013; Ruff et al., 2009; Siebner et al., 2009).

On the clinical application front, TMS has been approved by the FDA for the treatment of major depression (MDD) and obsessive-compulsive disorder (OCD), with other therapeutical applications on the way. Concurrent TMS/fMRI (Bergmann et al., 2021; Ruff et al., 2009) can be used to investigate the mechanism of action related to the immediate effects of the TMS stimulation as well as the cumulative therapeutic effects. For example, concurrent TMS/fMRI could be used to examine how TMS applied to a specific stimulation target influences activity across the entire network exhibiting aberrant excitability patterns in conditions such as OCD. Additionally, it can help determine how different stimulation targets relate to corresponding therapeutic outcomes (Duprat et al., 2025; Tik et al., 2023). This information could be used both to understand the mechanisms better and to improve the therapeutic outcome by fully personalized TMS protocols.

One of the main challenges of combining TMS with fMRI is that the instrumentation available to conduct these experiments (Mizutani-Tiebel et al., 2022) is still not fully optimized to achieve full brain coverage with high sensitivity. To overcome this challenge and accelerate the adoption of TMS/fMRI as a unique tool for “causal brain imaging”, we have designed, developed, and constructed the first of its kind RF Cap. The novel 26-channel receive-only RF coil array not only achieves full brain coverage with high SNR but also offers unrestrained TMS coil positioning and minimal loss of TMS power due to the small thickness of the cap. In general, our design will offer an easier and more comfortable setup to conduct these experiments with the possibility to apply the TMS at any position over the head and allowing a neuronavigation system to be used for both the positioning of the TMS coil and monitoring its movement during the fMRI acquisition. We expect that this will increase the reliability and reproducibility of this technique across a wide range of applications.

## 2 Methods

### 2.1 RF Cap design and construction

**Fig. 1.**
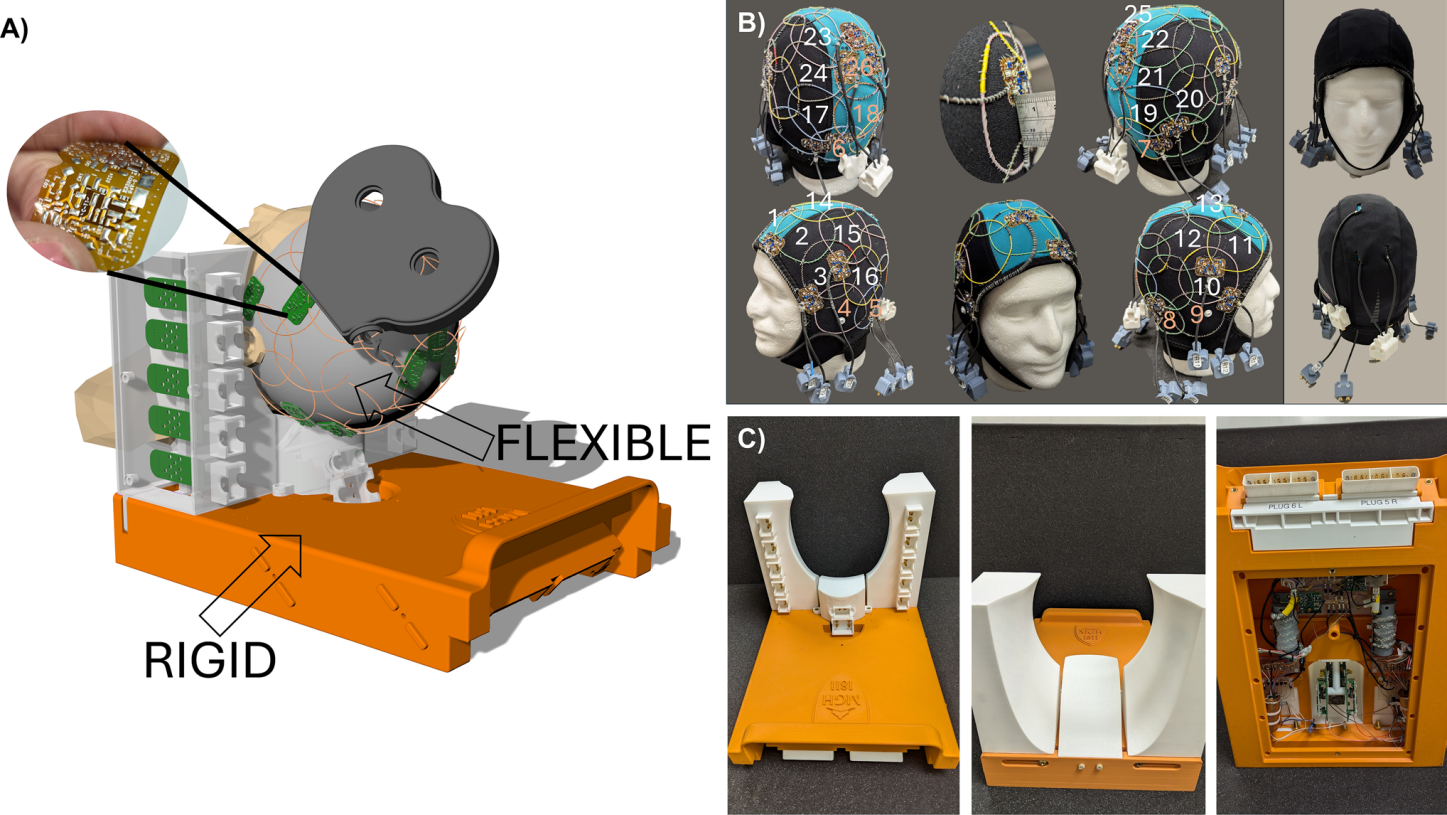
(A) 3D CAD Model of the RF Cap, consisting of the FLEXIBLE and the RIGID part. (B) FLEXIBLE Part. Left. Different perspectives of the FLEXIBLE part of the RF Cap. The numbers represent the RF elements distributed following the soccer ball layout. RF elements with red numbers were not included in the measurements of the detuning effect. One of the pictures shows a detail about the thickness of the elements used for the construction which is about 2 mm. Right. RF Cap fully covered for safety to be used with human subjects. (C) RIGID Part. Different views of the RIGID part where preamplifiers and electronics to interface the scanner are placed.

The RF Cap was designed to consist of two parts (see Fig. 1A). The flexible part includes the flexible RF coaxial cable loops, their flexible printed board circuits (PCBs) to interface them properly with the preamplifiers and the cables (not shown in 3D CAD Model). The rigid part includes (*i*) a holder for the neck and a fixation for the head that simultaneously holds the plugs for the cables coming from the RF loops and the preamplifiers and (*ii*) a base that holds all electronics to interface to the scanner directly (orange part).

#### 2.1.1 Flexible part: RF elements design and construction

The flexible part consists of a commercially available neoprene cap (2.5 mm) (Neoprene Diving Hood, Jecery, United Kingdom) with 26 flexible RF coaxial cable loops (Harpen, 1994; Nohava et al., 2021) sewn and distributed over the surface following a soccer ball layout (see Fig. 1B) to achieve the optimal geometric overlapping method whenever possible (Roemer et al., 1990). The cap is then covered with another commercially available thin neoprene cap (1.5 mm) (Thermoprene Sport Cap, Henderson, USA) with holes for the cables and plugs to isolate all electronics components from the subject (see Fig. 1B, left). A diameter of 7 cm was chosen for each RF loop to achieve a homogeneous coverage of the full head using 26 elements.

**Fig. 2.**
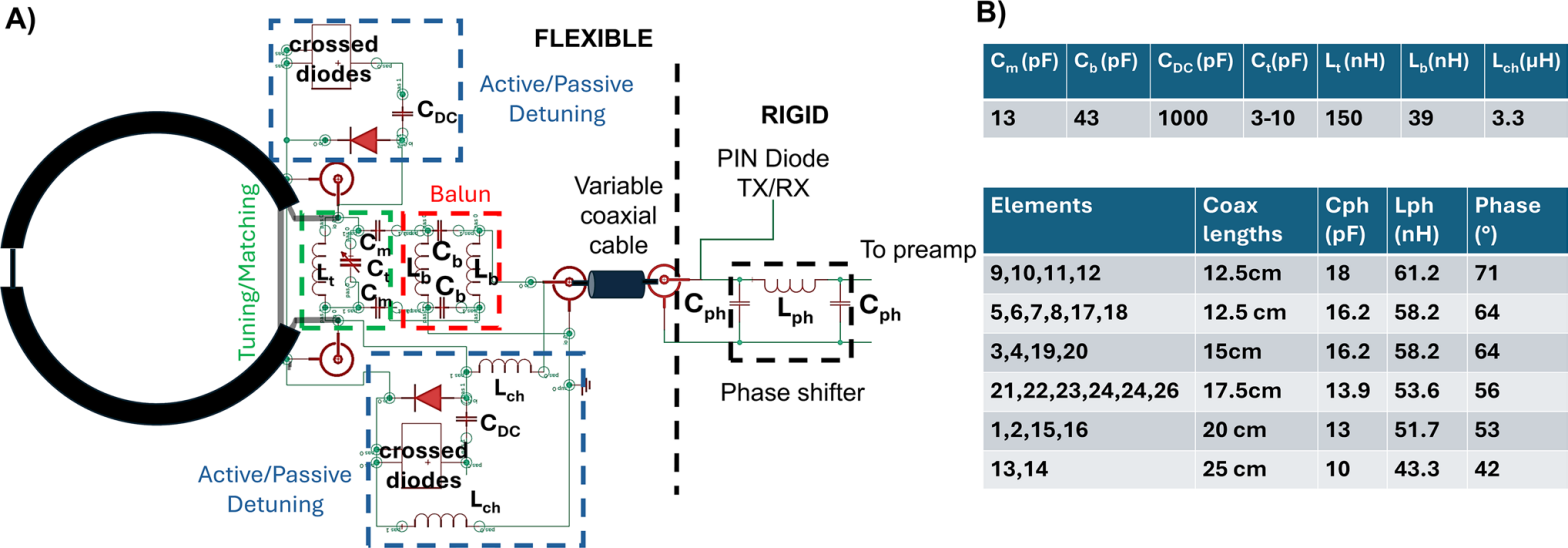
(A) Circuit diagram of the flexible RF coaxial cable element. (B) Table of passive elements’ values and cable lengths for each element and its corresponding phase shifter’s values.

The RF coaxial cable loops were constructed using thin coaxial cable (reclaimed Siemens coil cables, Z_0_ = 50 Ω) following the 1 turn/1 gap method described in Nohava et al. (2021). To obtain a reliable estimate of the impedance of these elements, we measured the input impedance of several prototypes using a vector network analyzer (EB071C, Agilent, Santa Clara, CA, USA). For this purpose, we soldered pin headers and calibrated the measurement to account for the effects introduced by the connection. Based on those results, we designed a tuning and matching network (see Fig. 2A) to finely tune the RF elements to be resonant at 123.25 MHz, Larmor frequency of the 3 T Skyra System (Siemens, Erlangen, Germany). The circuit matched the high impedance in resonance of the RF coaxial cable loops to an impedance of 18 Ω. The tuning/matching network was followed by a balun (balance to unbalance circuit) to both minimize common modes on the connecting cables and match the impedance to 50 Ω. The common-mode rejection ratio (CMRR) of the balun was measured with the previously described VNA using the three-port measurement technique reported by Skelton (2010). For this characterization, the balun printed on the flexible PCB was isolated, and the unbalanced port was matched to 18 Ω to minimize reflections at the VNA ports. The RF coaxial cable loops were interfaced in pairs using flexible PCBs (see Fig. 1A for details of the flexible PCB) to minimize the number of PCBs used. The part of the PCB where the elements for the balun were placed had a small ground plane on the bottom layer to increase electrical stability of the balun. The PCBs were carefully positioned to avoid common stimulation sites on the head, ensuring minimal thickness in those regions. The cables used to connect the PCBs with the preamplifiers are made of the same thin coaxial cable described above with variable lengths depending on their position on the RF Cap. The two cables per PCB are covered with heat shrink to minimize their spacing and connected to the preamplifier board using SMB connectors (female connectors on the cables side, male plugs on the rigid side). The resulting pair of cables were partially sewn to the cap to (*i*) achieve higher strain relief and (*ii*) to avoid conflicts with TMS coil positioning. To isolate the resonant RF coaxial cable loops from transmission, an active detuning mechanism was implemented in each loop using two PIN Diodes (Macom, MA4P7470F-1072T, Lowell, MA, USA) that will connect outer and inner conductors of the RF coaxial cable loops on both sides (see, active/passive detuning block in Fig. 2A) to eliminate the resonance as proposed in previous work (Nohava et al., 2021; Ruytenberg et al., 2020; Zhang et al., 2018). For equivalent circuits in receive and transmit mode, see Supplementary Figure S1. The diodes are biased through RF chokes (CoilCraft, 1008 CS, Cary, IL, USA) via the inner conductor of the connecting cable with the preamplifier boards. Additionally, we included a passive detuning mechanism using crossed diodes (Infineon, BAS 70-04 E6327, Neubiberg, Germany) and a DC blocking capacitor (Passive Plus LLC, 0505C, New York, USA).

We minimized the cable lengths connecting PCBs to the preamplifiers to optimize the cable losses. The cable lengths must be selected by considering the final phase shifter on the preamplifier board implemented to achieve preamplifier decoupling for each element (i.e., transform preamp input impedance to a high impedance at the coil drive point).

#### 2.1.2 Rigid part: Design and construction

The rigid part (see Fig. 1C) was designed using 3D CAD Software (Rhinoceros 7.0, McNeel&Associates, Seattle, USA) and 3D printed using acrylonitrile butadiene styrene (ABS for the base) and polylactic acid (PLA for holder and head’s fixation part). The neck holder was designed to have a slight inclination of 12.5 degrees to allow enough space for the TMS coil placement on the sides of the head and at the same time enable unobstructed connecting of the posterior cables to the plugs on the neck holder.

The base of the coil (in orange) hosts all coaxial cables from the preamplifier boards’ outputs placed on the side holders and on the neck holders. Some of the cables go through holes in the base that are partially covered afterward. The cables are arranged in two groups; the left bundle with six cables and the right bundle with seven cables (each cable carries two RF channels). Floating cable traps (Seeber et al., 2004) tuned to 123.25 MHz surround each of the bundles. The coaxial cables are connected using a PCB to another extra shielded bundle of coaxial cables connected at the end to an ERNI female connector. Each of the shielded bundles are wound around a 3D printed spiral form to facilitate the construction of an additional cable trap minimizing the common modes. Then, the bundles are connected to the two “direct connect” plugs (Siemens, Erlangen, Germany) using the ERNI male connectors.

Finally, we covered the sides of the fixation parts to isolate all electronic components from the environment and to protect the preamplifiers on the sides of the fixation system.

### 2.2 Bench measurements

Bench measurements were performed to adjust all components of the RF coil (i.e., tuning, matching, inter-element decoupling, and preamplifier decoupling), as well as to investigate the influence of the TMS coil when placed over the RF Cap using a double-loop probe according to the method proposed in Haase et al. (2000). The measurements were done using a vector network analyzer (EB071C, Agilent, Santa Clara, CA, USA).

As in our previous work (Navarro de Lara et al., 2023), we used a custom-made test rig to provide the required 3 V power supply for the preamplifiers and enable manual switching of the bias lines from forward (100 mA) to reverse bias (−10 V) to test the active detuning circuitry. Tuning and matching of the array elements were achieved by measuring the reflection coefficient S_ii_ for each channel. Additionally, we measured S_ij_ to guide the optimal position of the flexible RF coaxial loops over the cap. Preamplifier decoupling was tested with a double-loop probe according to the method proposed above. The measurements were performed after loading the RF coil with an in-house built anthropomorphic head phantom (Graedel et al., 2014).

### 2.3 MRI data acquisition and reconstruction for *phantom* measurements

#### 2.3.1 SNR performance and g-factor maps

For all the SNR performances and g-factor calculations, we acquired phantom images using the 2D GRE sequence listed in Table 1, saving k-space data and calculating the SNR maps for the optimal coil combination (Kellman & McVeigh, 2005) using in-house written MATLAB (Mathworks, Natick, MA, USA) scripts. The images acquired with different coils were co-registered using FSL (Analysis Group, FMRIB, Oxford, UK) and displayed in MATLAB.

**Table 1.** List of sequence parameters used for all measurements throughout the experiments.

MR Seq.	Parameters
2D GRE	2 mm in-plane resolution, slice thickness = 3 mm, 30 slices, matrix = 128 x 128, TE = 2.76 ms, TR = 5000 ms, FA = 90°
FA	2 mm in-plane resolution, slice thickness = 3 mm, 30 slices, TE = 2.22 ms TR = 19720 ms, matrix = 128 x 128, FA = 8°
B_0_	2 mm in-plane resolution, SL = 3 mm, 30 slices, TE1 = 4.66 ms TE2 = 7.12 ms TR = 755 ms, matrix = 104 x 120 and FA = 90°
MPRAGE	TR/TE/inversion time (TI) = 2530 ms/1.69 ms/1200 ms, FA = 7°, 176 slices, 1 mm isotropic, MA = 256 x 256, and BW = 651 Hz/pixel, GRAPPA = 2
EPI Phantom	TE = 35 ms, TR = 980 ms, 2.5 mm in-plane resolution, slice thickness = 2.5 mm, 26 slices, FA = 90°, MA = 80 x 80, MB = 2, GRAPPA = 3, 100 volumes, 20 ms gap
EPI *in vivo*	TE = 30 ms, TR = 2050 ms, 2 mm in-plane resolution, slice thickness = 2 mm, FA = 90°, 46 slices, MA = 100 x 100, MB = 2, 200 volumes
EPI *in vivo* concurrent TMS/fMRI	TE = 30 ms, TR = 1 s, 2.5 mm in-plane resolution, slice thickness = 2.5 mm with SMS = 5, GRAPPA = 2, FA = 64°, and 50 slices, 186 volumes

First, to compare the performance of the RF Cap with existing concurrent TMS/fMRI setups, we acquired phantom images placing the MRi-B90 TMS Coil (MagVenture, Farum, Denmark) over the left hemisphere of the phantom using a commercial fixation system (MagVenture, Farum, Denmark) in the scanner bore. For this first comparison, we acquired the data with the same phantom using (*i*) the RF Cap; (*ii*) the 1-ch transmit/1-ch receive Head Birdcage Coil that is the original coil used for full brain concurrent TMS/fMRI studies, and (*iii*) a combination of two 7-channel disk RF coils (Navarro De Lara et al., 2015), one on the stimulation site with the TMS attached and another on the contralateral site as described in a previous review article (Mizutani-Tiebel et al., 2022). Second, to get a benchmark of the quality of our data compared with common RF coils used in standard fMRI acquisitions, we collected data with the commercial 32-channel and 20-channel head coils (Siemens, Erlangen, Germany), even though such coils cannot be used in combination with TMS coils. Additionally, we calculated the g-factor maps for a middle transverse slice for each RF coil using the raw data and in-house MATLAB scripts following a previously published method (Pruessmann et al., 1999).

#### 2.3.2 B_0_ , B_1_^+^ maps and structural images

For an assessment of the constructed RF Cap in terms of B_0_ homogeneity, we acquired B_0_ maps of the phantom with the RF Cap using the vendor-provided B_0_ mapping sequence listed and described in Table 1. The acquired images were converted to Hz using MATLAB scripts and brain masked for clearer visualization of the data.

For the B_1_^+^ maps, we acquired data using the vendor-provided saturation-recovery flip angle (FA) mapping sequence (Chung et al., 2010) which parameters are detailed in Table 1.

A magnetization-prepared rapid acquisition gradient echo (MPRAGE) sequence was also performed to obtain anatomical images of the phantom with the sequence parameters listed in Table 1.

### 2.4 Interactions between the RF Cap and the TMS coil

To analyze the interactions between the TMS Coil and the RF Cap, we investigated three effects: (*i*) detuning effect on the resonance of the RF coaxial cable elements when the TMS is placed on the top of them, (*ii*) the effect on the stimulation efficacy due to the additional distance of the TMS to the brain, and *(iii*) the effect of the TMS coil on the image SNR when the TMS is placed over the RF Cap.

#### 2.4.1 Bench measurements to analyze detuning effects

The detuning effect was quantified (using the network analyzer described above) by measuring the reflection coefficient S_ii_ of most of the RF elements (except 4, 5, 6, 7, 8, 9, 18, and 26) when placing the TMS in different positions on the top of them. The RF elements that were tested were the ones on typical regions for TMS stimulation studies (elements excluded were the ones close to the ears, lower borders of the RF Cap, and in the posterior part). We moved the TMS coil across the RF elements from the left to the right and recorded the maximal detuning effect. The TMS coil was in physical contact with the RF Cap assuring minimum distance.

#### 2.4.2 Effect of RF Cap on stimulation efficacy

We determined the resting motor threshold (RMT) in three volunteers (2F/1M, 36.7 ± 12.4 years) with and without the RF Cap in place. All volunteers gave informed consent before participating in this study, which was reviewed by the IRB. For this procedure, we utilized an MRi-B90 TMS coil connected to a MagPro X100 stimulator (MagVenture, Farum, Denmark), with coil positioning guided by a neuronavigation system (Localite GmbH, Bonn, Germany). Motor evoked potentials (MEPs) from the *first dorsal interosseous* muscle were recorded during TMS using a BrainAmp ExG system (Brain Vision LLC, Garner, NC, USA). Initially, without the RF Cap, we manually identified the optimal coil position that consistently elicited the largest MEPs. The multiparametric Parametric Estimation by Sequential Testing (PEST) procedure (see, e.g., Borckardt et al., 2006) was then used to measure the RMT. Subsequently, we remeasured the RMT with the RF Cap in place (Fig. 1B, left). The neuronavigation system maintained consistent coil trajectory across both conditions, with position adjustments made only to accommodate the thickness of the RF Cap. The difference in motor threshold serves as a quantitative measure of stimulation efficiency loss, which our previous research has shown to be directly proportional to the additional distance created by the RF Cap (Navarro De Lara et al., 2015).

#### 2.4.3 Effect of TMS on SNR

To analyze this effect, we acquired 2D GRE images of the phantom using a sequence with parameters detailed in Table 1 under 2D GRE and calculated the SNR maps as described above for both cases, with and without the TMS coil over the RF Cap. For the conditions with TMS coil on the phantom, we used the same mechanical fixation system as described above. Data are presented as percentage gain or loss (the reference always being the measurement without the TMS coil) and was calculated as follows:

$$SNR_{\%}=\frac{\left(SNR_{TMS}-SNR_{NoTMS}\right)}{SNR_{NoTMS}}\;\cdot \;100\%.$$

### 2.5 Concurrent TMS/fMRI acquisition on a phantom

**Fig. 3.**
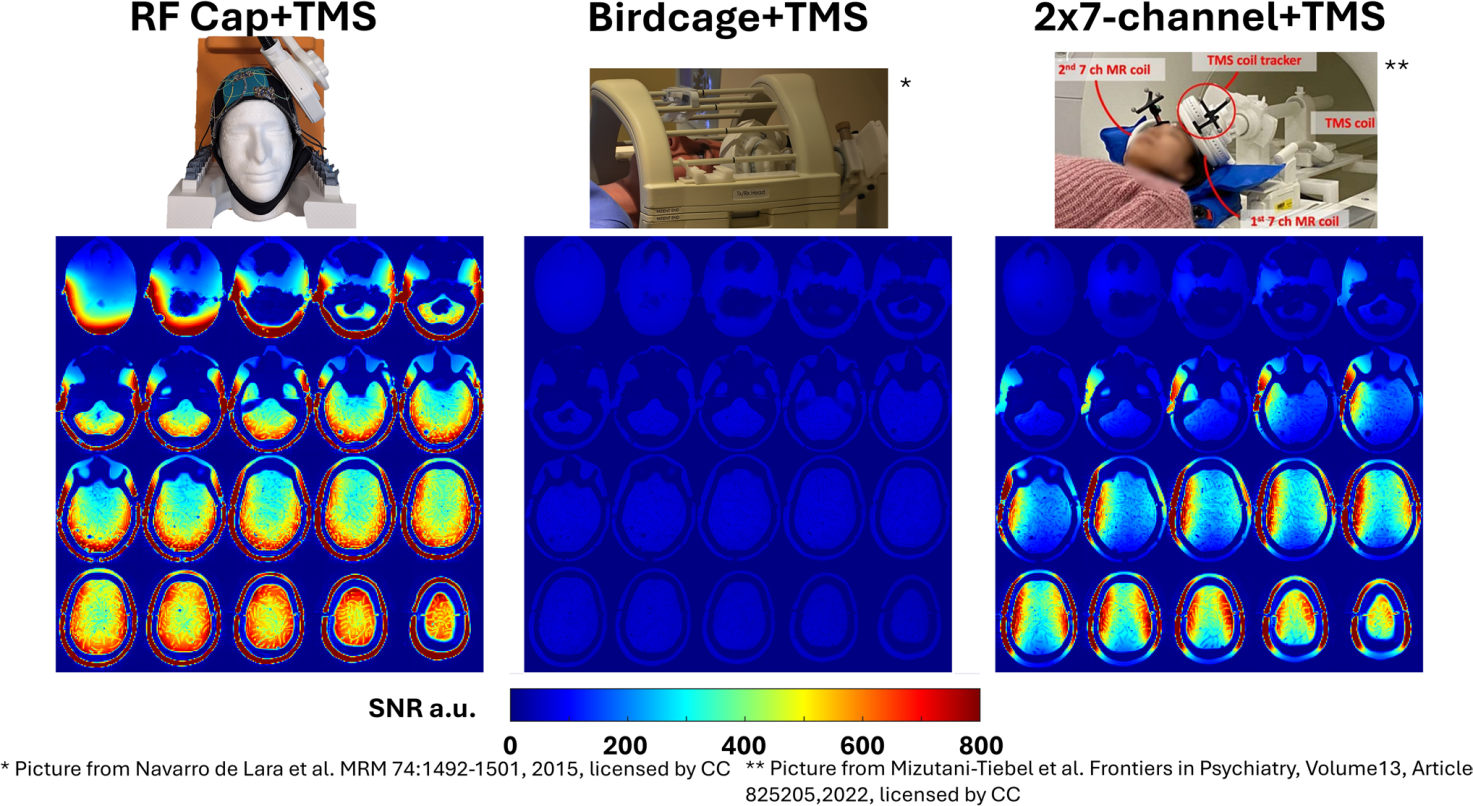
SNR maps acquired on head phantom comparing the RF Cap with existing setups for concurrent TMS/fMRI acquisitions. (Left) SNR map of the RF Cap with a TMS. (Center) SNR of a 1 TX/RX head Birdcage Coil with a TMS. (Right) SNR map of a combination of 2 x 7-channel thin disk RF coils, one placed on the stimulation site, and a second in the contralateral site.

To show the feasibility of the constructed RF Cap to acquire functional images while TMS pulses are applied, we performed a concurrent TMS/fMRI experiment on the phantom using the MRi-B90 TMS coil placed on the left hemisphere of the phantom (see Fig. 3 for the setup).

We acquired functional images of the phantom using state-of-the-art fast imaging 2D EPI sequence with parameters detailed in Table 1 under **EPI Phantom**. During the acquisition, a stimulation protocol was applied where TMS pulses were applied in 5 blocks (1 pulse per volume in the selected gap of 20 ms after volume acquisition) following a 10-volume period with no TMS pulses. The stimulation block was repeated three times. The intensity of the stimulator was chosen to be 40% of the maximal stimulator output (MSO). A simple pick-up loop was placed in the center of the bore to acquire detailed sequence timing data. The pick-up loop was connected to an oscilloscope placed in the console room using a long BNC cable through a waveguide. The temporal SNR was calculated as the mean of the time series signal divided by the standard deviation.

The TMS pulse triggering was implemented using Presentation software (Neurobehavioural Systems, Berkeley, CA, USA) synchronized with triggers produced by the scanner for each imaging volume. To record the trigger signals and the signal produced by the pick-up loop, we used a digital oscilloscope (RTB2004, Rhode&Schwarz, Munich, Germany).

### 2.6 *In vivo* imaging

Before we acquired imaging data *in vivo*, the RF Cap was subjected to a number of tests on the scanner to validate its performance and safety described in Keil et al. (2013). We measured the percentage of the RF power dissipated in the detuned receive array coil by comparing the RF body coil forwarded/reflected power levels required to achieve a 180° excitation in a phantom with and without the detuned array coil present. We considered a receive coil array validated, when the ratio of the absorbed power with the array versus without the array is between 0.8 and 1.2. We also tested the coil for heating. After switching off the specific absorption rate (SAR) monitor and the gradient stimulation monitor, we measured the temperature increase in the coil caused by RF transmit power being absorbed by the receive circuitry or heating by induced currents from the gradient switching. The detuned coil and phantom were scanned for 15 minutes with body coil transmitting at 150% of the usual SAR limit. The volunteer gave informed consent before participating in the study, and the imaging protocol was reviewed by the IRB.

#### 2.6.1 Echo-planar images (EPI) acquisition

To show the quality of functional images acquired with the constructed hardware, we acquired 2D EPI with parameters detailed in Table 1 under EPI *in vivo*. The subject was asked to keep his/her eyes open and remain relaxed and as still as possible (resting-state paradigm). The temporal SNR was calculated as the mean of the time series signal divided by the standard deviation.

#### 2.6.2 Structural images

A magnetization-prepared rapid acquisition gradient echo (MPRAGE) sequence was also performed to obtain anatomical images of the subject with the parameters detailed in Table 1 under MPRAGE.

#### 2.6.3 Concurrent TMS/fMRI acquisition

To demonstrate the high sensitivity of the RF Cap for *in vivo* imaging while applying TMS pulses in an interleaved fashion, we performed a short concurrent TMS/fMRI session on a healthy volunteer, targeting the primary motor cortex (M1). The protocol consisted of five stimulation blocks, each including five rTMS bursts, with each burst delivering four pulses at 20 Hz. These bursts were interleaved with the imaging sequence, occurring once per second, followed by a 20-second rest period. In total, 100 pulses were delivered in a single run (see Fig. 8A, bottom, for a schematic of the protocol). Stimulation was applied at 110% of the resting motor threshold (RMT). The RMT, determined inside the scanner bore using an MR-safe electromyographic (EMG) device (Bittium, Oulu, Finland), was 74% MSO. TMS coil placement was guided by an MR-compatible neuronavigation system (Localite, Bonn, Germany).

Imaging was performed with the parameters detailed in Table 1 under EPI *in vivo* concurrent TMS/fMRI. This setup enabled full-brain volume acquisition in 623 ms at 2.5 mm isotropic resolution. A 377 ms temporal gap followed each volume acquisition, during which rTMS bursts were delivered—starting 120 ms after the previous volume ended and ending 107 ms before the start of the next acquisition. All volumes were acquired without distortion produced by the TMS pulses. FMRI data were preprocessed using FSL (version 7.4.1; FMRIB Software Library, Oxford, UK) and FreeSurfer (https://surfer.nmr.mgh.harvard.edu/), following standard pipelines for task-based fMRI analysis. Motion correction was performed using FSL’s **MCFLIRT**, followed by correction for susceptibility-induced distortions with **TOPUP**, integrated with FreeSurfer routines. Functional images were then registered to the participant’s T1-weighted anatomical image using boundary-based registration via **BBRegister**, part of Freesurfer. A temporal high-pass filter was applied to remove low-frequency artifacts. Statistical analysis was conducted using a general linear model (GLM), with task regressors convolved with a canonical gamma hemodynamic response function. No spatial smoothing was applied.

## 3 Results

### 3.1 RF Coil properties

The input impedance of the RF element prototypes at the Larmor frequency ranged from 1.5 to 4.6 kΩ for the resistive component (R) and from 2.1 to 3.5 kΩ for the reactive component (X). All RF elements of the coil were tuned and matched to 123.25 MHz achieving a |S_11_|< -15 dB. Coupling between the elements (|S_ij_|) was less or equal -18 dB. The CMRR of the balun was measured as 26.7 dB.

**Fig. 4.**
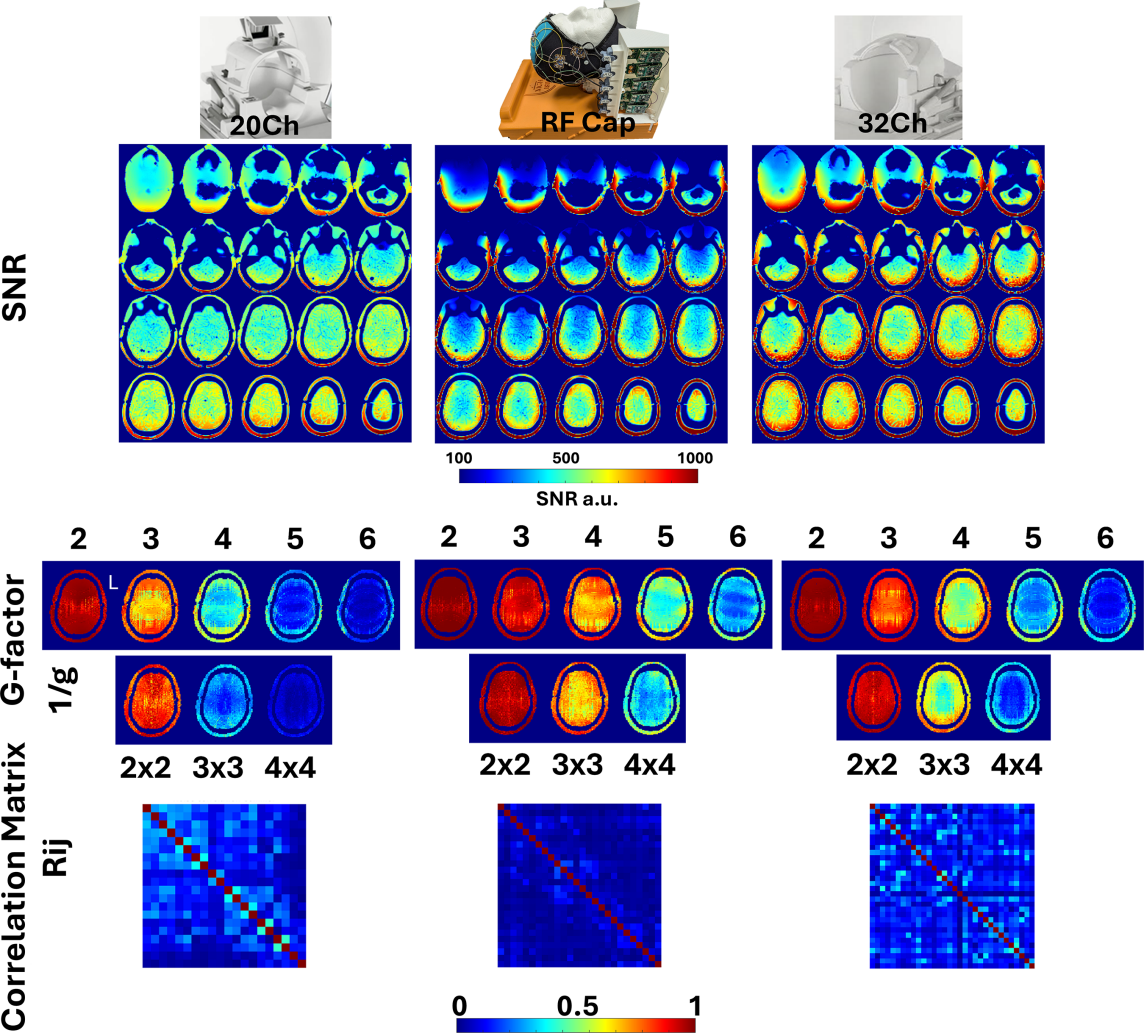
Performance comparison with commercially available RF coils at 3T. (Top) SNR maps of the commercial 20-channel RF coil, the RF Cap, and the commercial 32-channel RF Coil. (Center) G-factor maps of the commercial 20-channel RF coil, the RF Cap, and the commercial 32-channel RF Coil. The maps are presented for one dimension acceleration factors 2, 3, 4, 5, and 6 and for two dimensions acceleration factors 2 x 2, 3 x 3 and 4 x 4. (Bottom) Correlation matrix of the commercial 20-channel RF Coil, the RF Cap, and the commercial 32-channel RF Coil.

The active detuning efficiency was tested with the switches on the test rig. Using our double decoupled probe, we observed a flat line with no resonance at all when the active detuning mechanism was engaged. We also observed that elements required less than -20 dBm power from the network analyzer to avoid activating the passive detuning mechanism. These results were verified during our safety test, where we observed less than 20% change in the scanner’s transmit adjust RF power when the RF Cap was present in the bore compared with when it was absent (with identical phantom positioning in both cases). Inter-element noise correlation values ranged from 0.1% to 20.2%, with an average of 3.6%. No significant changes were observed in the correlation matrix when we measured noise with or without the TMS coil. The correlation matrix is shown at the bottom of Figure 4.

### 3.2 SNR performance comparison with existing concurrent TMS/fMRI setups

The SNR maps for the existing setups and the RF Cap are shown in Figure 3. The RF Cap shows an SNR at least 4 times higher than the SNR of the Birdcage Coil at the center of the phantom and 10–16 times higher on the brain surface of our phantom. In the case of the two 7-channel disk RF coils, the RF Cap shows an SNR up to 7 times higher than the SNR achieved with the two disk coils in the regions where neither coil directly covers the brain. In the center of the brain, the SNR of the RF Cap is between 20% to about 200% higher depending on how inferior the slice is. However, in the regions directly under the disk coils, the RF Cap shows 10–25% less SNR than the disk coils. This is observed for “cortical regions” of our phantom up to maximal 2 cm and is limited to central region of the disk coil.

### 3.3 SNR performance comparison with commercial RF coils and g-factors maps

The SNR maps for the RF Cap and the two other commercially available head RF coils are shown on the top of Figure 4. Compared with the commercial 32-channel RF coil, the RF Cap shows about 65% of the SNR achieved with the commercial 32-channel RF coil at the center of the brain. In frontal and inferior positions, the RF Cap achieves only a 45% of the SNR of the commercial 32-channel RF coil due to the lack of elements placed both in the neck and over the forehead. In cortical regions, the results vary depending on position. The RF Cap offers between 10 and 20% less SNR to similar SNR. Compared with the commercial 20-channel RF coil, the RF Cap shows about 65–70% of the SNR at the center of the brain. At frontal and inferior positions, the SNR of the cap is 58% of the SNR of commercial 20-channel RF coil. In cortical regions, the RF Cap offers up to 20% more SNR depending on the position, consistently outperforming the commercial 20-channel RF coil. As a reminder, the commercial 20-channel RF coil has not only a lower number of channels but also larger dimensions than the commercial 32-channel RF coil (20-channel inside RF coil dimension; 21 cm x 26 cm x 28 cm vs. 32-channel inside RF coil dimensions; 19.5 cm x 23 cm x 22.5 cm), and, therefore, it is expected to be less sensitive than the commercial 32-channel RF coil.

The g-factor maps are shown at the center of Figure 4 for each of the three evaluated RF coils. The g-factor maps for the RF Cap show superior values than the commercial RF coils for most of the brain except for the very inferior slices covering part of the neck, where the RF Cap has limited or no coverage. The average 1/g-value over the slice for the acceleration factor R = 3 is 0.91 for the RF Cap, while for the commercial 32/20-channel RF coils, these values are 0.84 and 0.73, respectively. For higher acceleration factors like 4, this value for the RF Cap decreases to 0.75 and for the commercial 32/20-channel RF coils, the values decrease to an average 0.6 and 0.44, respectively. For two-dimensional acceleration 3 x 3, the RF Cap delivers an averaged 1/g-factor of 0.71, while the commercial 32/20-channel RF coils only achieve an average 0.58 and 0.25, respectively.

The correlation matrix calculated for each coil with the noise acquired for the SNR calculations is shown under the g-factor maps at the bottom of Figure 4.

Taken together, the SNR of the RF Cap at the cortex is comparable with the commercially available RF coils, however, for deeper and frontal regions, it achieves about 45% of the 32-channel and about 70% of the 20-channel RF coil’s SNR. In the case of the g-factor maps, the RF Cap performs comparably with the 32-channel and outperforms clearly the 20-channel RF coil’s parallel imaging capabilities.

### 3.4 B_0_, B_1_^+^ maps and structural images

**Fig. 5.**
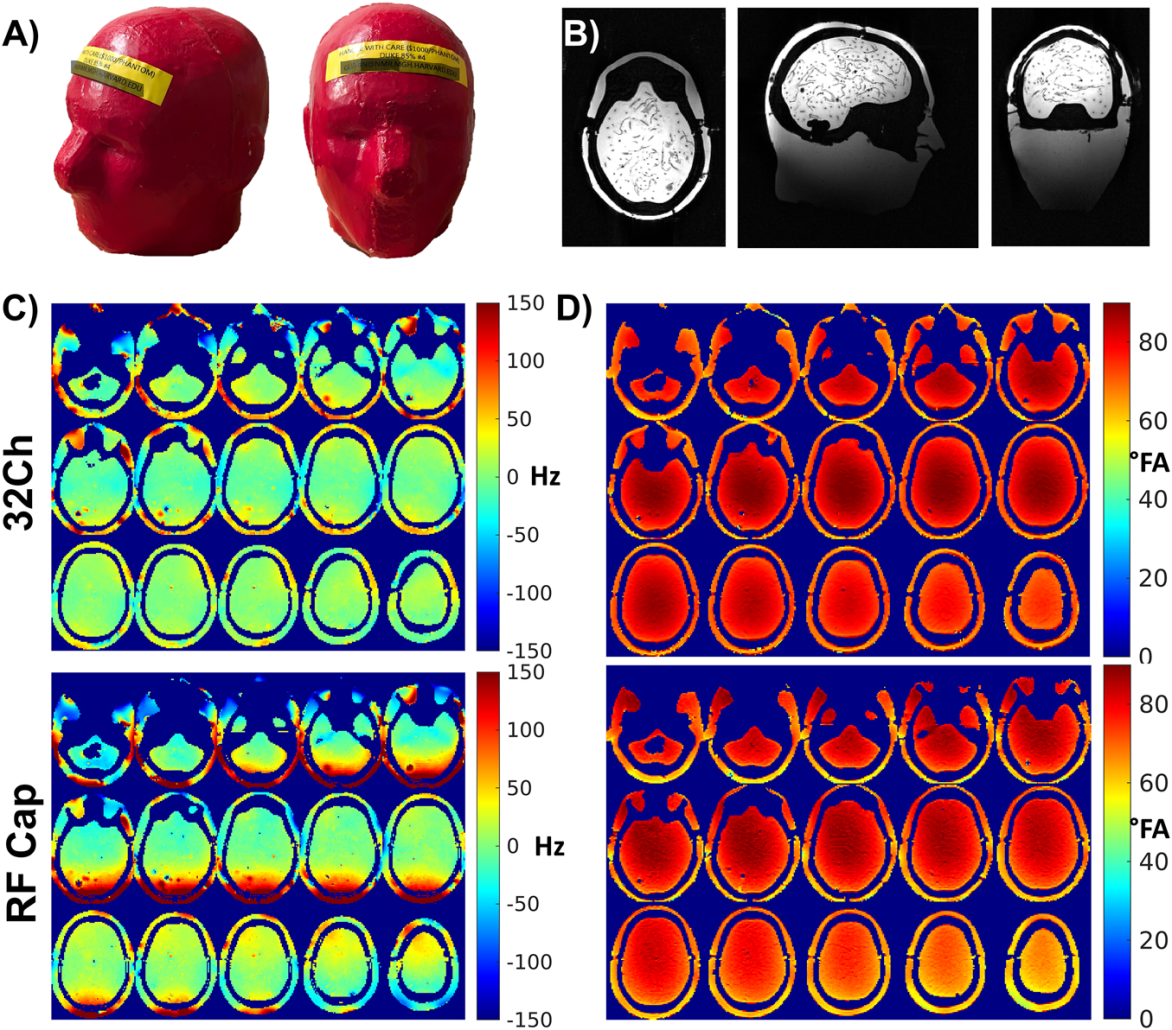
B_0_, B_1_^+^ maps and structural images using the RF Cap. (A) Picture of the anthropomorphic phantom used in this study for the SNR assessment. (B) T1/MPRAGE images of the phantom acquired with the RF Cap. (C) B_0_ maps acquired with 32-channel commercially available RF coil and the RF Cap. We see B_0_ offsets for the RF Cap at the rear part of the cap, possibly due to the cables. (D) B_1_^+^ map of the phantom acquired with the commercial 32-channel RF coil and the RF Cap.

Figure 5A shows pictures of the phantom we used for all measurements. Its structural images obtained with the RF Cap are shown in Figure 5B.

The B_0_ map obtained on the phantom using the RF Cap is shown at the bottom of Figure 5C. The values obtained ranged from -55 to 204 Hz in the brain. The inhomogeneities are especially strong in the posterior part of the phantom, where three cables and three PCBs are joined. For comparison, we show the B_0_ map obtained using the commercial 32-channel RF coil on the same phantom at the top of Figure 5C.

The B_1_^+^ map obtained on the phantom using the RF Cap is shown at the bottom of Figure 5D. The flip angles achieved with the RF Cap especially in the more superior slices are slightly lower than the flip angles obtained using the commercial 32-channel RF coil, shown at the top of Figure 5D. The minimal flip angles in the upper slices in the brain were about 54° compared with 67° obtained with the 32-channel. Otherwise, it follows the typical brightening in the center of the phantom as expected.

### 3.5 Interactions between the RF Cap and the TMS coil

#### 3.5.1 Detuning effect on the flexible RF coaxial cable elements

The measured mean maximal detuning effect over all the analyzed RF elements (marked with white numbers in Fig. 1B) was found to be 1.88 MHz ± 0.4 MHz, which as percentage change is 1.5% ± 0.32%. The obtained value is almost half the value reported in previous studies (Navarro De Lara et al., 2015). The observed lower detuning values of the coaxial cable loops compared with copper loops are indicative of smaller SNR losses when the TMS coil is placed over the phantom due to this effect.

#### 3.5.2 Effect of the RF Cap on stimulation efficacy

The mean increase in the RMT was found to be 7.3% points with a standard deviation of 1.2% points. This value is in accordance with the estimated loss of magnetic field due to the thickness of the RF Cap of about 3–4 mm.

#### 3.5.3 Effect of TMS on SNR

**Fig. 6.**
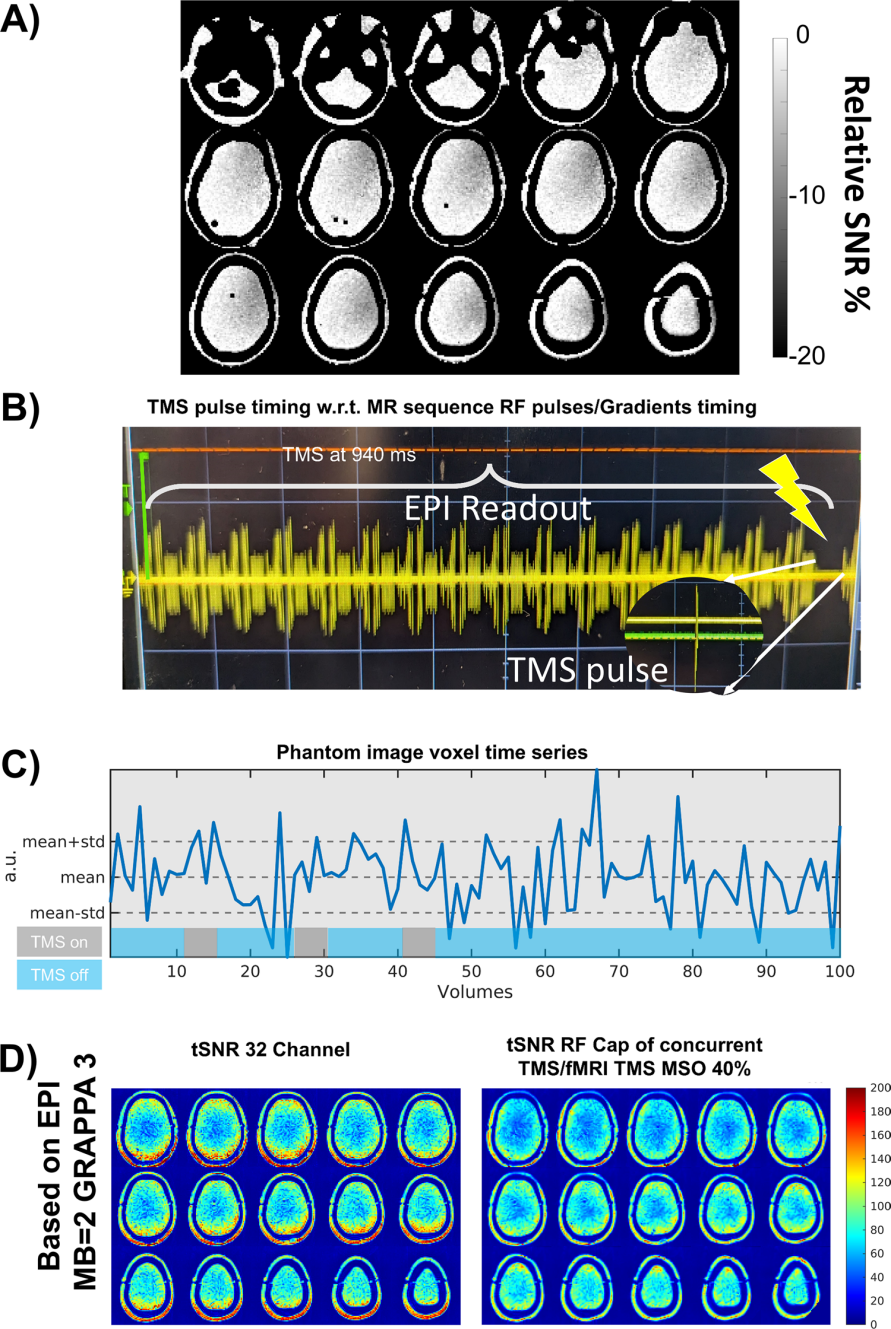
Results of the concurrent TMS/fMRI experiment over a phantom using the RF Cap. (A) Relative SNR percentage change of the RF Cap when the TMS is placed over it. (B) Screenshot of the timing synchronization of the TMS pulse with the MR sequence to avoid image artifacts in the images. Signal acquired with the pick-up loop. (C) Phantom image time series of a voxel close to the TMS target. The lower blocks represent the stimulation protocol, the volumes with and with no stimulation pulses. (D) Comparison of temporal SNR of the RF Cap and the commercial 32-channel RF Coil using the selected fMRI sequence (MB = 2 and GRAPPA = 3).

The percentage loss of SNR when placing a TMS coil over the RF Cap is shown in Figure 6A. The loss ranges from 5% to 20%, being highest close to the cortex and decreasing for slices that are deeper and further away from the stimulation coil.

### 3.6 Concurrent TMS/fMRI acquisitions

We successfully performed a concurrent TMS/fMRI phantom experiment using the RF Cap. There are no visible artifacts due to the stimulation in the acquired images and we obtain full brain coverage with high tSNR. The summary of the results of the concurrent TMS/fMRI acquisition is shown in Figure 6B–D.

First, the synchronization of the TMS with the sequence was successfully implemented as described in the Methods section as shown in Figure 6B. We did not observe any artifacts in the acquired volumes before or after the TMS pulses were applied. The stimulation protocol is shown in Fig 6C. Second, the tSNR of the acquired functional images was calculated and is shown together with the tSNR obtained with the commercial 32-channel RF coil in Figure 6D. It is interesting to note that for superior slices, the tSNR of the RF Cap reaches similar values to the 32-channel RF coil. The effects are specific to the used sequence as high acceleration factors have been used.

### 3.7 *In vivo* acquisitions

**Fig. 7.**
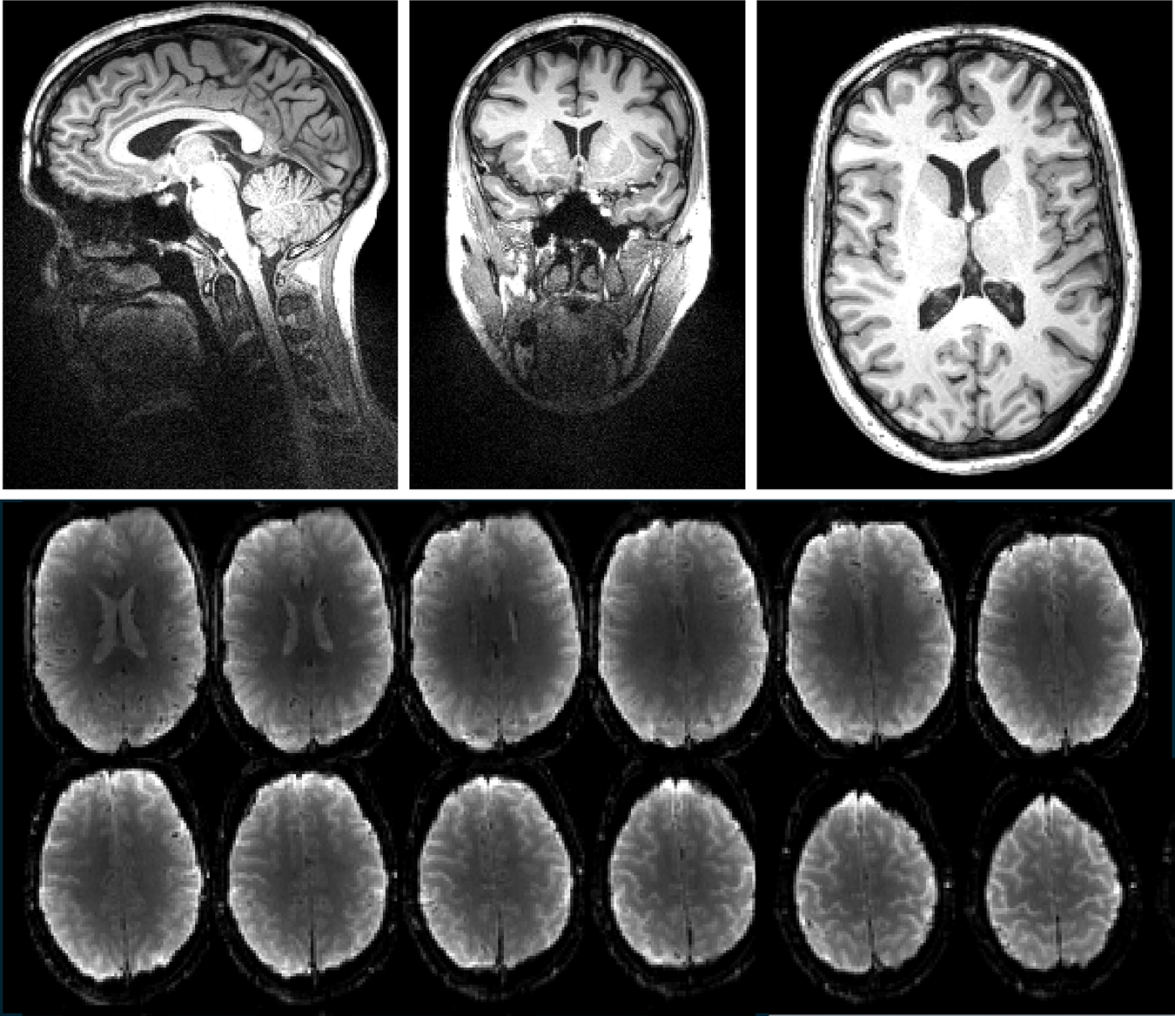
First in vivo images acquired with the RF Cap. (Top) T1w/MPRAGE in vivo images acquired using the RF Cap (with no TMS coil present). Main central planes are shown: sagittal, coronal, and transversal. (Bottom) In vivo full brain functional EPI images (46 slices in total, not all shown) acquired with the RF Cap with a 2 mm isotropic resolution and a TR = 2050.

The first in vivo acquisitions of the RF Cap are shown in Figure 7. Structural and functionals images clearly demonstrate the capabilities of the RF Cap to achieve full brain coverage with high imaging quality.

#### 3.7.1 Concurrent TMS/fMRI acquisitions

**Fig. 8.**
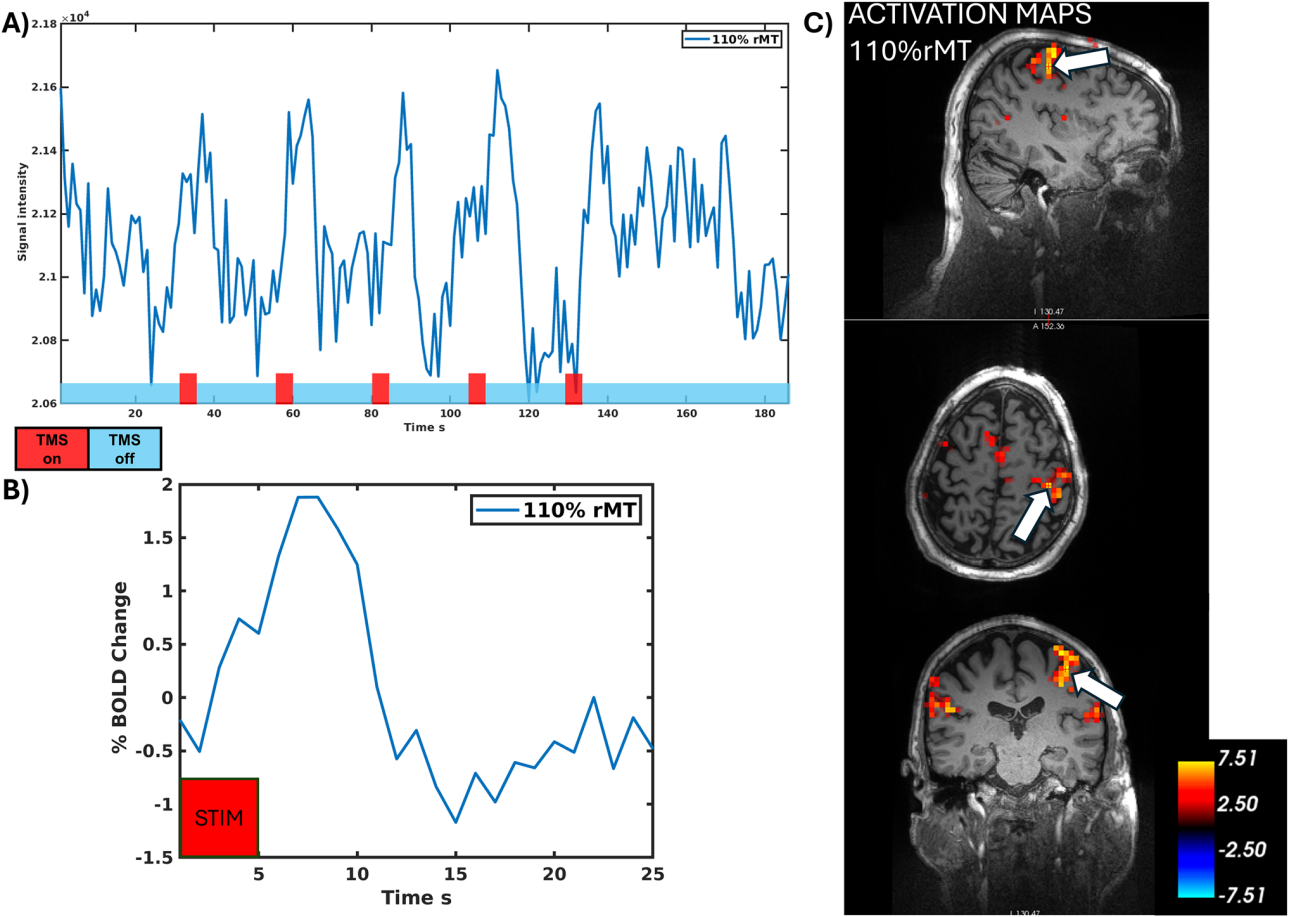
First in vivo concurrent TMS/fMRI experiment with the RF Cap. (A) Time series of the voxel on M1 showing local effects of TMS stimulation (indicated by the white arrow on the activation maps). (B) Bold signal time courses averaged over stimulation blocks. (C) Activation maps for the single run over the anatomical image acquired with the RF Cap.

The summary of the results of the first *in vivo* concurrent TMS/fMRI experiment using the RF Cap is shown in Figure 8. In Figure 8A, we show the time course from the single run at the voxel indicated by the white arrow on the activation maps, with very clear stimulation effects visible in the raw data. Figure 8B presents the average BOLD signal changes across all stimulation blocks. Notably, the post-stimulation undershoot in the hemodynamic response is also clearly visible despite averaging only five blocks, demonstrating the high sensitivity of our RF Cap to measure brain activity. Figure 8C shows the activity maps at 110% intensity condition revealing some remote activations, suggesting that our method is sufficiently sensitive to measure whole-brain network-level target engagement using BOLD fMRI.

## 4 Discussion

In this article, we present for the first time a whole-head RF coil capable of allowing concurrent non-invasive brain stimulation using TMS coils and the acquisition of high-quality structural and functional magnetic resonance images of the human brain. To enable this powerful combination of brain stimulation and imaging, we have designed a flexible and extremely thin tightly fitting RF Cap. The flexible RF Cap conforms to the individual subject’s head shape, therefore, maximizing the filling factor and minimizing thickness, allowing brain stimulation to be administered with best possible electromagnetic field efficiency.

We presented the SNR comparison with existing concurrent TMS/fMRI setups. The results show a drastic boost not only in the achievable SNR and brain coverage, allowing higher spatial and temporal resolution, but also potentially increasing the subject comfort significantly. Having the ability to place the TMS coil freely over the head with unhindered adjustment of the position/orientation will greatly facilitate the use of a neuronavigation in conjunction with a mechanical coil holder. We consider that these features are essential to transform this challenging but powerful multimodal TMS/MRI technology into an “easy to use,” controlled and robust neuroscience tool and pave the way for future clinical applications.

We designed the RF Cap with a strong focus on wide practical applicability: the combination of concurrent TMS and fMRI to achieve causal brain imaging across the whole brain. For this reason, the coaxial cables interfacing the RF elements with the preamplifiers needed to be (*i*) of minimal length to minimize losses, (*ii*) sewn on the cap to avoid being in the way of the TMS coil, and (*iii*) have minimal thickness to avoid efficiency losses in the TMS administration. For this reason, we implemented a lattice balun in the PCB to minimize possible common modes on the interface cables. For these baluns, we incorporated a ground plane in the bottom layer to increase stability of the coil electrical parameters. These design decisions might have an impact on the B_0_ and B_1_^+^ maps of the RF Cap as shown in the results. The inhomogeneity increase in the B_0_ map is especially high in the posterior part of the RF Cap due to the presence of three cables and the confluence of three PCBs. A possible strategy to mitigate this effect would be positioning the PCBs interfacing elements 21/22 and 23/24 (see Figure 1B) more on the lateral side and away from each other. Additionally, in this scenario the cables would not cross, and the cable length would remain the same, minimizing cable losses. Having some distance between the PCBs and their cables will increase the B_0_ homogeneity. For the B_1_^+^, the lower FA achieved on the more superior slices could be explained by the interaction of the transmit field with the ground planes on the PCBs. Implementing the changes proposed above would also improve the B_1_^+^ efficiency as the three PCBs will be more distant from each other. However, the changes in power transmitted obtained when measuring with the body coil in the presence/absence of the RF Cap were less than 20% following our safety protocols.

In the past 10 years, there have been extensive technical development efforts in flexible RF coil designs for MR imaging. From screen-printed flexible coils (Corea et al., 2016), RF coils built using conductive silver-coated thread (Vincent & Rispoli, 2020), or using braided conductors on elastic fabrics (Nordmeyer-Massner et al., 2012), resonators formed by liquid metal in polymer tubes (Port et al., 2020) to the use of coaxial cables (Zhang et al., 2018) or twisted pair (Maravilla et al., 2022) to build the RF elements. In our case, the choice was the flexible RF coil technology based on self-resonant coaxial cable coils (Harpen, 1994; Ruytenberg et al., 2020; Stensgaard, 1996; Zhang et al., 2022). We chose this approach due to the intrinsic low coupling with neighboring coils reported in previous publications (Mollaei et al., 2020) and confirmed by our results. This property is ideal for ensuring the decoupling performance of the RF coil and, therefore, the g-factor maps that are stable independently of the head size and form. At the same time, the flexible RF coaxial cable loops experience fewer detuning effects and eddy current effects than standard copper loops when TMS coils are placed close to them (Navarro De Lara et al., 2015, 2023), which is critical for the TMS-fMRI application of the presented hardware. This can be explained by the conservative E-fields being largely confined between the coaxial center conductor and shield and, therefore, experiencing less E-field coupling to TMS coils and to the body load. This effect was also explained in Ruytenberg et al. (2020), and relies in our opinion on the lower resistive coupling coefficient (k_e_) of these types of RF loops to the sample compared with the standard copper loops. To conclude, the flexible RF coaxial cable loop couples less with the sample with the negative consequence of achieving less SNR than standard copper loops; however, the effects of the TMS on the imaging are less profound than when using standard copper loops as shown by our results. Specifically, for the application of the RF Cap in conjunction with TMS, we cannot readjust the tuning depending on the TMS coil position to re-gain some of the lost SNR like proposed in previous work (Navarro De Lara et al., 2015). Overall, our results suggest this property is still advantageous as the detuning effect is less pronounced than in previous approaches (Navarro De Lara et al., 2015, 2023), making the imaging quality consistent across different stimulation target locations. Other technologies such as the twisted-pair elements as RF coils have been proposed for flexible RF coil design (Maravilla et al., 2023), though no data about the robustness of these elements when a TMS is placed nearby have yet been reported.

We successfully conducted a concurrent TMS experiment with an anthropomorphic head phantom and on a volunteer. We were able to acquire full brain functional images interleaved with the application of TMS pulses with high temporal and spatial resolution without artifacts. We should also point out one of the most important practical advantages of our constructed instrumentation: it will significantly shorten the preparation time of this rather challenging experiment, maintaining the comfortability for the subjects, as the TMS coil positioning is not hindered at all by the RF coil. Additionally, the low inter-element coupling of the RF Cap is reflected in low g-factor maps, allowing leveraging parallel imaging methods to accelerate the acquisition and shorten the overall duration of the scans. The presented tSNR maps of the RF Cap demonstrate this capability when using a functional imaging sequence with SMS-factor of 2 and GRAPPA-factor of 3 and achieving a comparable tSNR with a commercial 32-channel head coil.

To advance our knowledge of how distributed brain networks process information, we need to develop causal brain imaging techniques that enable “perturb and measure” type paradigms that can be readily applied in large-scale human studies. The results obtained with the RF Cap presented here show high-quality data that we can acquire while stimulating brain regions with very little limitations in coil positioning and in a well-controlled setups with possibility to utilize neuronavigation and high degree of subject comfort. In addition to conventional single-channel setups, the developed RF Cap can be used in combination with recently developed multi-channel TMS systems (Navarro de Lara et al., 2021), which will advance further causal brain imaging by enabling stimulation of multiple regions simultaneously or shifting the target stimulation without coil movement. The presented RF Cap is the beginning of a new technology for head RF coils for combined acquisitions that solves many of the existing challenges. However, there are still some practical aspects that will have to be investigated such as SNR and g-factors maps stability for different head sizes and robustness of the approach for high patient/volunteer workflow.

## Data and Code Availability

The scripts to calculate SNR maps and g-factors can be accessed at https://github.com/lunade/MATLAB_SNR_gfactors_tools.

## Supplementary Material

Supplementary Material
