# Supplementary material for: The RF Cap: A 26-channel flexible RF coil cap for optimized concurrent TMS/fMRI experiments at 3T
Source: Imaging Neurosci (Camb). 2025 Oct 14;3:IMAG.a.922. doi: 10.1162/IMAG.a.922 (PMC12521982; doi:10.1162/IMAG.a.922)
Supplement: Supplementary Material [file IMAG.a.922_supp.pdf]

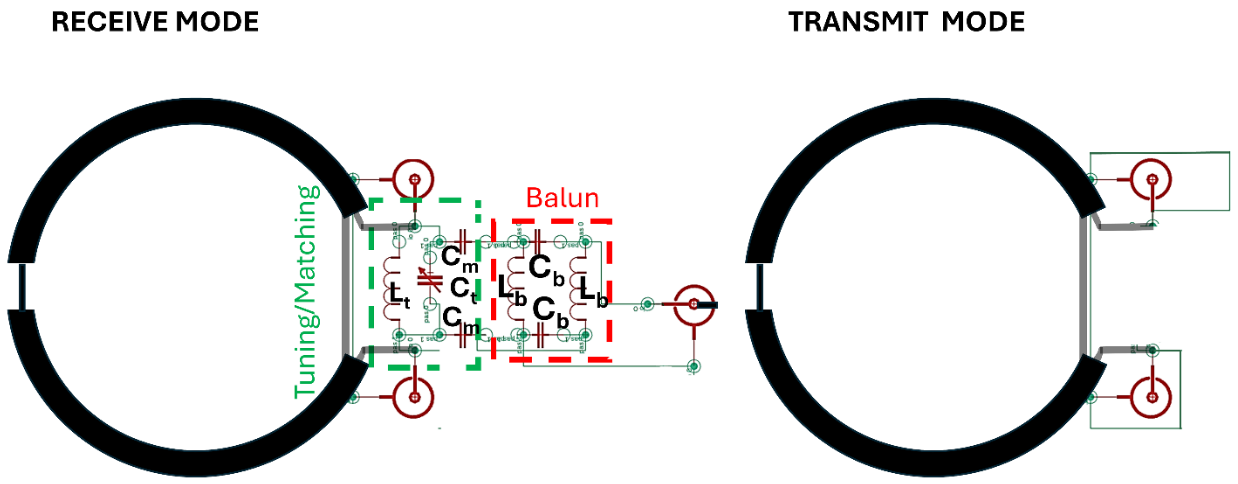

Figure S1. Equivalent Circuits in receive and transmit mode. Left) In receive mode, the coil "sees" only the tuning/matching circuitry and the balun. The active/passive detuning circuits are inactive as the diodes are not forward biased. **Right)** In transmit mode, both sides of the inner conductor in the feed port are connected to their outer shield, eliminating the self-resonance of the element.
